# Multimodal Pilot Evaluation of a Hyaluronic Acid Infraorbital Filler Using Precise, Multipositional, 3-Dimensional Imaging Quantification, Patient-Reported Outcomes, and Anatomic Cadaveric Assessments

**DOI:** 10.1093/asjof/ojae086

**Published:** 2024-10-07

**Authors:** Stephanie E Honig, Yoshiko Toyoda, Jane N Ewing, Mehdi S Lemdani, Zachary Gala, Chris Amro, Alexander T Wilson, Robyn B Broach, Ivona Percec

## Abstract

**Background:**

Despite the high demand of filler in the infraorbital area, there remains debate on injection practices, precise anatomical placement, and hyaluronic acid (HA) filler behavior.

**Objectives:**

We aimed to contribute to the clinical and anatomic understanding of infraorbital HA injection through a prospective patient injection study in combination with a cadaveric analysis.

**Methods:**

Patients were injected with Volbella XC (JUVÉDERM, Allergan Aesthetics, an AbbVie Company, Irvine, CA) into the tear trough region by a single experienced aesthetic plastic surgeon. Over a 90-day period, precise undereye volumetric measurements using 3-dimensional photogrammetry (VECTRA-M3, Canfield Scientific, Inc., Fairfield, NJ) and patient-reported outcomes (PROs; FACE-Q) were collected and analyzed relative to 2 pretreatment severity scales. Juvéderm Vycross (Allergan Aesthetics, an AbbVie Company, Irvine, CA) and Restylane NASHA (Galderma, Lausanne, Switzerland) products were injected into the infraorbital and malar region in 6 cephalus specimens and evaluated with regards to the anatomic injection location with and without common clinical physical manipulations.

**Results:**

Eleven patients participated with a 100% retention rate. Infraorbital HA volume maintenance was 70% to 81% at 30 days and 50% to 70% at 90 days. Significant improvement was noted in the eyes, overall facial appearance, and cheekbones (*P* < .05) with FACE-Q outcomes, irrespective of pretreatment severity. In the cadaver examination, we observed differences in the anatomic locations occupied by Juvéderm and Restylane products as well as in behavior after physical manipulation between gel types.

**Conclusions:**

Volbella XC effectively augments undereye volume to diminish infraorbital hollowing as measured over a 90-day period with significantly improved PROs. Enhanced knowledge of the behavior of Volbella XC and other HA fillers in this sensitive anatomic region will lead to improved patient outcomes.

**Level of Evidence: 4 (Therapeutic):**

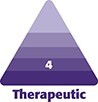

Soft tissue filler injections are the second most common nonsurgical cosmetic procedure performed in the United States, with over 3.2 million procedures reported in the 2022 Plastic Surgery Statistics Report, of which about 76% are hyaluronic acid (HA)-based fillers.^1^ Juvéderm Volbella XC (Allergan Aesthetics, an AbbVie Company, Irvine, CA) is an HA gel filler that consists of a low HA concentration tightly crosslinked HA using Vycross (JUVÉDERM, Allergan Aesthetics, an AbbVie Company, Irvine, CA) technology. It was originally approved for injection into the lips for lip augmentation and correction of perioral rhytids. It has since been FDA approved for injection into the infraorbital region (IOR) to improve the appearance of undereye hollows, or infraorbital hollows (IOHs), in adults over the age of 21.^2^ More recently, Restylane Eyelight (Galderma Laboratories, Fort Worth, TX), an HA filler with different rheological properties from Volbella, was FDA approved for use in this region.^3^

The tear trough and lid–cheek junction demonstrate among the earliest and most noticeable signs of aging because of thin periorbital skin and underlying support tissues, as well as loss of volume causing shadowing of the IOR.^4,5^ Thus, rejuvenation of this area is commonly requested by patients early in the aging process. Fabi et al conducted a Phase III clinical randomized controlled trial in which patients with moderate or severe IOH were treated with Volbella XC and followed up with touch-ups over the course of a year, yet precise data on volumetric changes regarding in patients without touch-ups, as well as the role of the orbicularis muscle is lacking.^6^ Further, debate on injection practices and precise anatomical placement, due in part to controversies on the anatomic understanding of the region and its delicate nature, confound the literature and clinical experience.^7,8^ We herein aim to conduct a pilot study to (1) investigate the precise volumetric response in patients with small volume Volbella XC injection to the IOH utilizing 3-dimensional (3D) photogrammetry, (2) better understand how Volbella XC injection may contribute to overall patient satisfaction using patient-reported outcomes (PROs) and validated infraorbital pretreatment severity scales, and (3) contribute to the anatomic understanding of infraorbital filler injection placement through a cadaveric examination using Juvéderm Vycross and Restylane NASHA (Galderma, Lausanne, Switzerland) products with different constitutions and manipulation techniques.

## METHODS

We performed a multimodal evaluation, which consists of 3 different methods to address the research question: (1) 3D imaging for quantitative analysis, (2) PROs for qualitative analysis, and (3) cadaveric analysis. The prospective patient analysis was performed to analyze 3D volumetrics of the IOR and its relationship to preseverity treatment scales and PROs. The use of other imaging modalities, such as ultrasound and computed tomography (CT) scans, was not selected because of their inconsistent and imprecise capacity to quantify small changes in volume in the IOR and our extensive experience with 3D photogrammetry technology. The accompanying cadaveric analysis was performed to enhance our anatomic understanding of infraorbital filler injection placement using Juvéderm Vycross and Restylane NASHA products with different rheology, constitutions, and manipulation techniques. A hemifacial comparison of these products was conducted ex vivo, as Restylane was not an FDA-approved drug for the treatment of the IOH at the time of the study and could not be performed in vivo.

### Prospective Patient Injection Pilot Study

We conducted a single-center study from January 6, 2023 to April 17, 2023, on females between 22 and 65 years old who consented to and received infraorbital filler injections by a single plastic surgeon with extensive expertise in facial injections. Patients were compensated $15 for each follow-up visit and questionnaire completed. Exclusion criteria included: (1) prior filler in tear trough/midface, (2) prior cosmetic facial surgeries above the cheekbones, (3) prior facial trauma (ie, orbital fracture), (4) filler or neurotoxin injection within the past 12 months, and (5) pregnancy or breastfeeding.

The study was granted approval by the institutional review board (Protocol #852212) at the University of Pennsylvania Health Systems and was registered at clinicaltrials.gov (NCT# 05694286). Written consent was provided, by which the patients agreed to the use and analysis of their data. This study was conducted in accordance with the ethical guidelines of the 1975 Declaration of Helsinki, obtaining informed consent and following good clinical practice.

### Tear Trough and Infraorbital Orbital Hollowing

The tear trough appears as a natural subtle groove along the inferior margin of the lower eyelid. The exact anatomic contributions to the tear trough remain somewhat disputed among anatomists. The length of the groove extends from the medial canthus to the medial third of the lower eyelid, before it blends into the lid–cheek junction, which runs parallel to the infraorbital rim.^6,9^ IOH results from a loss of volume causing shadowing and darkness of the IOR. The IOH can extend from the medial canthus to the lateral canthus along the infraorbital rim.

### Injection Technique

Each patient received up to 1 cc per hemiface of Juvéderm Volbella XC (15 mg/mL) to the tear trough region (Supplemental Figure 1, Video). After skin cleansing, all injections were performed medially to laterally at the supraperiosteal plane of the infraorbital rim to regions of hollowing with the manufacturer-provided 32 G 0.5-inch needle and syringe. The amount and location of injections varied between patients to achieve ideal correction. Minimal pressure was applied for contouring only if needed without massage. No topical products or cooling was applied pre- or posttreatment. Restylane Eyelight (Galderma, Lausanne, Switzerland) was not FDA approved or commercially available at the time of the study; however, it was used for the Video for an expanded description of the injection technique. Adverse events, including hematoma or significant immediate or delayed swelling, were recorded.

### 3D Volumetric Assessments

**Figure 1.**
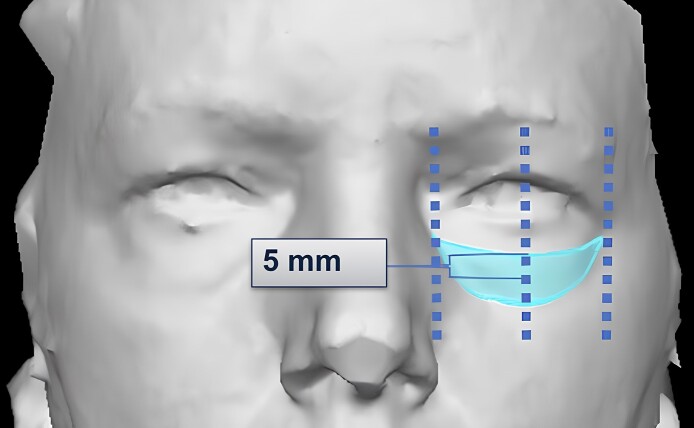
Area of interest for VECTRA 3D imaging. Width of the area was defined by the distance between the medial and lateral canthi. Height of the area was 5 mm above and below the tear trough and lid–cheek junction. The peak curvature of the area occurred mid pupil.

The 3D photogrammetry of the frontal view was taken with VECTRA M3 (Canfield Scientific, Inc., Fairfield, NJ) for volumetric analysis. The area of interest was determined by the following parameters: (1) the width of the area was defined by the distance between the medial and lateral canthi, (2) the height of the area was 5 mm above and below the tear trough and lid–cheek junction, and (3) the peak curvature of the area occurred mid pupil (Figure 1). All VECTRA analyses were completed with imaging software (VECTRA Analysis Module [VAM], Canfield Scientific, Parsippany, NJ). A standardized step-by-step protocol was created with VECTRA representatives to ensure consistent techniques in aligning the areas of interest to its reference point to mitigate variability between subjects and the quality of the photograph. All photographs were registered using each subject‘s preinjection photograph as reference.

Using immediate postinjection volume for reference, as derived from the preinjection volume, changes in right and left infraorbital volume were independently measured at 3 additional time points: 14, 30, and 90 days. Tissue displacement factor (TDF) was calculated by dividing immediate postinjection volume by injected HA filler volume (cc). The effective baseline volume (EV) was calculated as the percent of HA when dividing the 14-day postinjection volume by immediate postinjection volume. Volumes were measured in 3 positions: open frontal gaze, closed eyes, and upward gaze. Temporal volume changes were visualized using a color scale in which the gradient transitions from blue to green, yellow, or red to signify decreasing volumes.

### Validated Patient-Reported Outcomes

The validated PRO measure, FACE-Q, was administered at all time points (0 pre- and postinjection, 14 days, 30 days, and 90 days). FACE-Q questionnaire consisted of 9 domains: eye, lower eyelids, cheek, cheekbones, expectations, age, facial appearance, psychosocial distress, and psychosocial function. Each domain had 7 to 10 items that were calculated and converted to a Rasch score (0-100, with higher scores indicating better outcome). Specifically, the lower eyelids domain assessed patient perception of their under eye for the following: excess fat, excess skin, puffiness, lines, wrinkles, how old the area made you look, and how tired the area made you look.

### Pretreatment Severity Scales

Right and left IOH prior to treatment was evaluated by the senior surgeon using 2 validated instruments: Allergan Infraorbital Hollow Scale (AIHS) and Tear Trough Rating Scale (TTRS). The AIHS is a validated scale for objective and reproducible comparisons of IOH before and after dermal filler injection.^10^ The TTRS is another validated method of evaluating tear trough deformities that measures depth of trough, degree of hyperpigmentation, prolapse of nasal fat pads/pockets, and lower eyelid skin rhytidosis.^11^

Correlations between the AIHS and the FACE-Q domains were computed, analyzing left and right under eye independently from each other. The process was repeated between the TTRS and FACE-Q domains. When controlling for asymmetry of the scores (ie, AIHS *R* = 2, AIHS L = 1), the difference between the right and left undereye rating scores was measured. Correlation with the difference in scores and FACE-Q domains that mentioned “symmetry” was evaluated. All correlations were completed using the Kendall Rank Correlation Coefficient.^12,13^ Statistical significance was set at a *P*-value <.05. Data analysis was performed in Excel 365 and R-Studio (version 4.3.1).^14^

### Cadaveric Analysis

**Figure 2.**
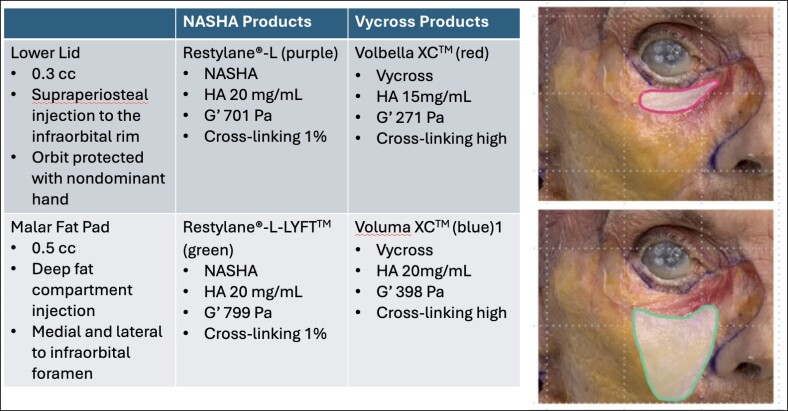
Filler injection by product and location.^15^

Institutional approval for cadaveric study was obtained. Six fresh cadaveric cephalus specimens were used for filler injection and dissection. All fillers were dyed with food coloring in a consistent fashion. The Juvéderm products Volbella XC (tear trough, red) and Voluma XC (malar, blue) were used together in hemifaces (*Juvéderm pair*), and Restylane products Restylane-L (tear trough, purple) and Restylane-LYFT (malar, green) were used together in separate sets of hemifaces (*Restylane pair*; Figure 2).

All filler injections were performed by the senior author as follows: 0.5 cc of filler was injected into the malar region in the deep medial fat compartment superficial to the maxilla with attention to avoiding the infraorbital foramen by palpation and anatomic landmarks. In the tear trough region, the orbit was protected with the nondominant hand, and 0.3 cc of filler injected on the infraorbital rim periosteum. Injections were performed using the same technique the author employs on subjects. In 2 cadavers, the hemiface was injected with the original constitution of filler product, and the other was injected with blended filler (1 cc of HA blended with 0.2 cc of saline). In 2 cadavers, the hemifaces were massaged after injection and compared with those which remained unmanipulated.

All hemifaces were then dissected from medial to lateral in the subcutaneous plane and between the superficial and deep facial fat compartments by a trained single senior plastic surgery resident. All photographs and videography were taken by a single author. All hemifaces were evaluated by all authors.

## RESULTS

### Prospective Patient Injection Pilot Study

#### Demographics

Eleven subjects participated, producing a sample size of 22 undereye regions to be evaluated. Enrolled participants were all female, 90% White, with an average age of 43.2 ± 15.2 (range, 24.8-63.8; Table 1). When evaluated by the senior author, subjects had a baseline average grade of 2.3 on the AIHS and 6.9 on the TTRS scale, both indicating moderate-to-severe undereye hollowness. All participants had bilateral Volbella XC injections (up to 1 cc each side) conducted with identical technique. There was a 100% retention rate over the 90-day period. There were no adverse events, including hematoma or significant immediate or delayed swelling.

**Table 1.** Patient Characteristics

Demographic categories	*n* = 11
	*n* (%)
Age	
Mean (SD)	43.2 (15.2)
Median (Q1, Q3)	39.6 (29.9, 58.5)
Min-Max	24.8-63.8
Female	11 (100)
Caucasian/White	10 (90)
Non-Latino or non-Hispanic	11 (100)
BMI	
Mean (SD)	26.3 (6.1)
Median (Q1, Q3)	24.9 (22.0, 29.1)
Min-Max	19.1-36.9
Smoking history	
Never	10 (90)
Unknown	1 (10)
Injection characteristics	
Bilateral injection	11 (100)
Juvéderm Volbella	11 (100)
Average volume (mL) of right infraorbital injection (SD)	0.88 (0.16)
Average volume (mL) of left infraorbital injection (SD)	0.88 (0.31)
Allergan Infraorbital Hollow Scale	*n* = 22
Grade 1	5
Grade 2	9
Grade 3	4
Grade 4	4
Tear Trough Rating Scale, mean (SD)	6.9 (1.9)

SD, standard deviation.

#### Tissue Displacement Correction Factor Calculation

The TDF was calculated for Volbella XC HA to the IOH to define the immediate volumetric tissue response of a given Volbella XC HA volume on the infraorbital tissue when injected at the periosteal plane. Immediate postinjection volume was measured and divided by the actual injected HA volume to generate the TDF. The TDF was calculated to be 0.29 and 0.28, respectively, for the right and left lower IOR with frontal gaze in repose. With the activation of the orbicularis oculi (eyes closed), the TDF was 0.3 and 0.23 for the right and left, respectively. With maximum relaxation of the orbicularis oculi (upward gaze), the TDF was 0.18 and 0.21 for the right and left, respectively (Table 2). There was no statistically significant difference in TDF by laterality.

**Table 2.** Median Tissue Displacement Factor and Effective Baseline Volume

Infraorbital region	TDF	EV
Open, frontal gaze	*n* = 11	
Right	0.29	0.93
Left	0.28	0.97
Closed	*n* = 7	
Right	0.3	0.96
Left	0.23	0.90
Upward gaze	*n* = 7	
Right	0.18	0.94
Left	0.21	0.81

Tissue displacement correction factor (TDF) was calculated by dividing postvolume injections (cc) by injected hyaluronic acid filler volume. Effective baseline volume (EV) was calculated by dividing 2-week volume by immediate postvolume injection.

#### Effective Baseline Volume Calculation

Effective baseline volume (EV) after HA injection was defined as the 14-day postinjection volume, to factor in the presence of lidocaine, resolution of acute inflammatory period and edema/bruising, as well as tissue accommodation to the placed HA. The percent of HA maintained in the EV was calculated by dividing the 14-day postinjection volume by immediate postinjection volume. The EV was calculated to be 0.93 and 0.97, respectively, for the right and left lower IOR with frontal gaze in repose. With the activation of the orbicularis oculi, the EV was 0.96 and 0.91 for the right and left, respectively. With maximum relaxation of the orbicularis oculi, the EV was 0.94 and 0.81 for the right and left, respectively (Table 2). There was no statistically significant difference in EV by laterality except when eyes were closed (*P* = .03).

#### Volume Maintenance Calculation

The EV, effective baseline volume, at 14-day postinjection was used to analyze percent HA volume maintenance over 30 and 90 days for all 3 eye views. Infraorbital HA volume maintenance at 30 days was found to be 70% upon frontal gaze, 80% upon eye closing, and 81% upon upward gaze. At 90 days, maintenance was found to be 50% upon frontal gaze, 70% upon eye closing, and 63% upon upward gaze. There was no statistically significant difference in volume maintenance by laterality. See Table 3 for median volume change and percent filler maintenance.

**Table 3.** Median Volume Change and % Filler Maintenance

Infraorbital region and views	Median volume (cc)	Median volume change (cc)	Volume maintenance
Open, frontal gaze (right)	*n* = 11		
Post	0.25	—	1.00
14 days	0.23	−0.01	0.93
30 days	0.19	*−0*.*04*	0.85
90 days	0.14	*−0*.*11*	0.59
Open, frontal gaze (left)	*n* = 11		
Post	0.23	—	1.00
14 days	0.23	−0.01	0.97
30 days	0.14	*−0*.*09*	0.70
90 days	0.12	*−0*.*12*	0.50
Open, frontal gaze (both)	*n* = 22		
Post	0.23	—	1.00
14 days	0.23	*−0*.*01*	0.97
30 days	0.14	*−0*.*07*	0.70
90 days	0.12	*−0*.*11*	0.50
Closed (right)	*n* = 7		
Post	0.41	—	1.00
14 days	0.40	−0.02	0.96
30 days	0.32	*−0*.*07*	0.80
90 days	0.29	*−0*.*08*	0.72
Closed (left)	*n* = 7		
Post	0.31	—	1.00
14 days	0.23	*−0*.*03*	0.90
30 days	0.21	*−0*.*06*	0.79
90 days	0.21	*−0*.*10*	0.68
Closed (both)	*n* = 14		
Post	0.31	—	1.00
14 days	0.23	−0.02	0.92
30 days	0.21	−0.07	0.80
90 days	0.21	−0.09	0.70
Upward gaze (right)	*n* = 7		
Post	0.26	—	1.00
14 days	0.24	−0.01	0.94
30 days	0.22	*−0*.*05*	0.82
90 days	0.17	*−0*.*08*	0.66
Upward gaze (left)	*n* = 7		
Post	0.22	—	1.00
14 days	0.20	*−0*.*03*	0.81
30 days	0.18	*−0*.*05*	0.80
90 days	0.14	*−0*.*09*	0.63
Upward gaze (both)	*n* = 14		
Post	0.22	—	1.00
14 days	0.23	*−0*.*01*	0.91
30 days	0.21	*−0*.*05*	0.81
90 days	0.21	*−0*.*09*	0.63

The values given in Italics are statistically significant at *P* < .05.

#### Visualization of Volume Change

**Figure 3.**
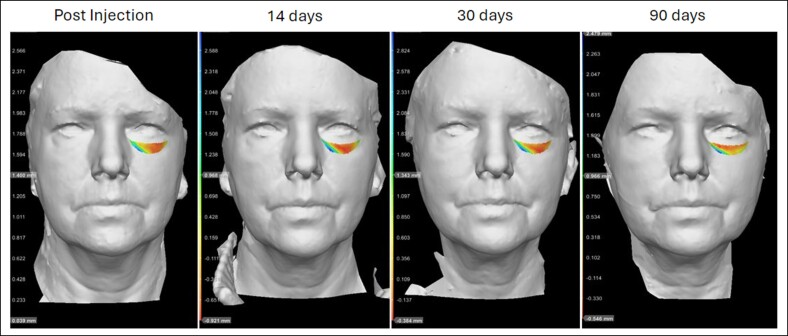
3D photogrammetry with color gradient including areas of interest was defined at 4 different time points (postinjection, 2 weeks, 4 weeks, and 12 weeks). All photographs were registered to preinjection photographs.

We visualized volume changes over time across participants through color gradient and area of interest. The color gradient from blue to yellow over time suggests HA volume decrease begins medially before it spreads laterally over time (Figure 3).

#### Patient-Reported Outcomes

**Figure 4.**
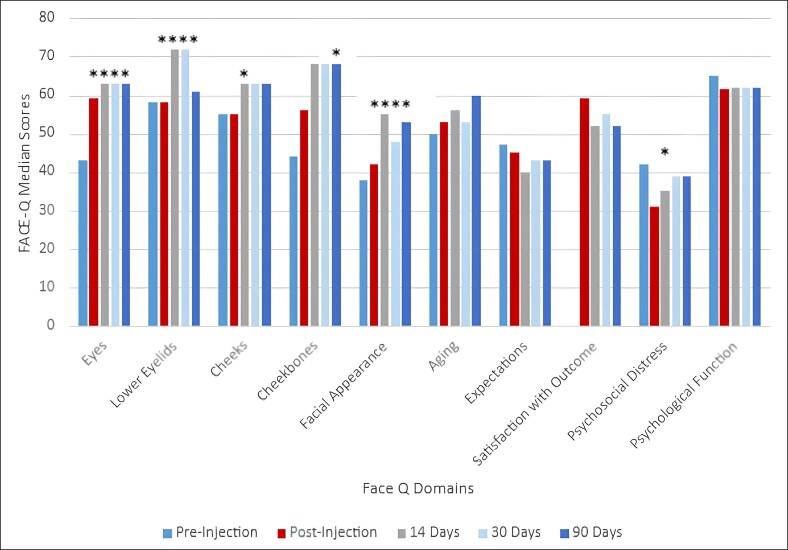
FACE-Q domain scores at post, 14 days, 30 days, and 90 days in comparison with preinjection scores. * means significant difference in scores when compared to pre-injection scores.

When comparing FACE-Q responses between postinjections (immediate, 14 days, 30 days, and 90 days) to preinjection, there was a significant improvement in how participants rated their eyes, lower eyelids, and overall facial appearance (*P* < .05; Figure 4). Significant positive change was noted in perception of cheekbones at Day 90 compared with preinjection (*P* = .007).

#### Validated Pretreatment Severity Scores

AIHS and TTRS pretreatment severity had little to no correlation with FACE-Q improvement. Within all calculated correlations, the greatest correlation was between AIHS (right or left) and “Aging” and TTRS (right) and “Aging.” However, correlation was weak (AIHS R: *r* = −0.483; AIHS L: *r* = −0.431; TTRS R: *r* = −0.345) and did not reach significance (AIHS R: *P* = .056; AIHS L: *P* = .086; TTRS R: *P* = .166). There was significant moderate correlation between TTRS (left) and “Aging” (TTRS L: *r* = −0.675, *P* = .006; see Table 4). When controlling for asymmetry of the pretreatment severity scales and the bilateral FACE-Q domains consisting of “symmetry,” no statistically significant correlation was observed.

**Table 4.** Correlation Between Pretreatment Severity Scores to the FACE-Q Domains

Pretreatment severity scales	Allergan infraorbital hollow scale				Tear trough rating scale			
	Right		Left		Right		Left	
FACE-Q domains	Coefficient	*P*-value	Coefficient	*P*-value	Coefficient	*P*-value	Coefficient	*P*-value
Eyes	−0.02	.933	0	1	−0.02	.934	−0.02	.935
Lower Eyelids	0.11	.676	0.04	0.87	.31	0.219	0.02	.935
Cheeks	0.02	.933	−0.02	.93	−0.10	.681	−0.27	.290
Cheekbones	−0.02	.933	−0.11	.68	−0.06	.806	−0.18	.464
Facial Appearance	0.39	.145	0.29	.27	0.20	.449	−0.07	.802
Aging	−0.48	.056	−0.43	.09	−0.35	.166	−0.68	.006
Expectations	0.10	.674	0	1	0.25	.324	0.33	.192
Psychosocial Distress	−0.04	.867	−0.04	.87	0.04	.870	0.22	.371
Psychosocial Function	0.25	.320	0.12	.63	0.06	.808	−0.02	.936

### Cadaveric Analysis

**Figure 5.**
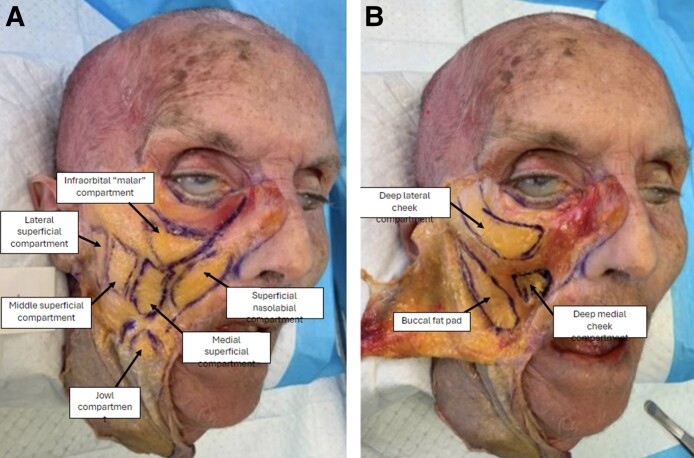
Cadaveric cephalus specimen dissected to reveal the (A) superficial fat pads and (B) deep fat pads.

All fresh cadavers were in excellent condition, and the subcutaneous tissue, superficial fat, and deep fat compartments were dissected in clear planes (Figure 5). After HA injection, on dissection of the lower eyelid in the standard, nonmodified, specimens, Volbella XC was observed to be on the periosteum of the infraorbital rim and along the orbicularis retaining ligament (ORL), the targeted site of injection. Volbella XC transitioned superficially into the suborbicularis oculi fat and the subcutaneous plane. In contrast, Restylane-L, also observed on the infraorbital rim periosteum, did not spread into the adjoining tissues, and remained as a distinct product on the infraorbital rim and ORL.

**Figure 6.**
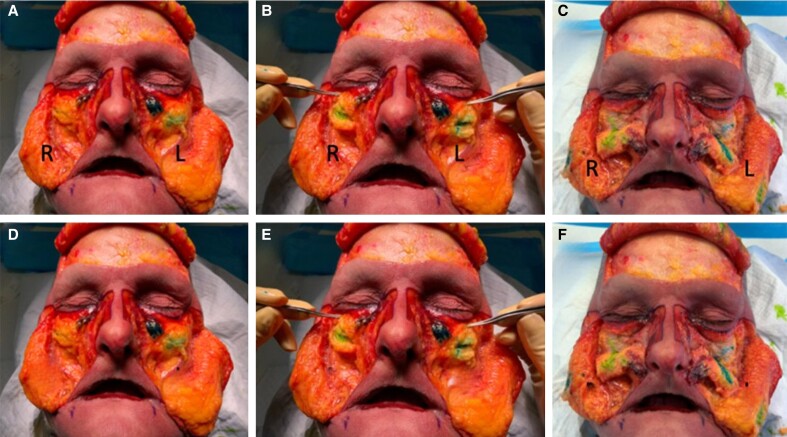
Cadaver injected with Restylane-L in the tear trough and restylane-LYFT in the malars on the right, Juvéderm volbella XC and Juvéderm voluma XC on the left after (A) subcutaneous dissection, (B) SMAS dissection, and (C) deep fat compartment dissection. (D) Cadaver injected with Juvéderm Volbella XC in the lower lid and Juvéderm Voluma XC in the malars; Filler product was nonblended on cadaveric right (R) and blended (0.2) on cadaveric left (L). (E) Cadaver injected with Restylane-L in the lower lid and Restylane-LYFT in the malars. (F) Filler product was nonblended on cadaveric right (R) and blended (0.2) on cadaveric left (L).

On dissection of the malar region after injection, Voluma XC was observed to be in the deep medial cheek compartment, the targeted site of injection. Voluma XC transitioned superficially into the superficial medial cheek and superficial nasolabial fat compartments and subcutaneous fat. In contrast, Restylane-LYFT remained largely within the deep medial cheek compartment without spreading into the superficial compartments (Figure 6A-C).

Manipulation of Juvéderm products Volbella XC and Voluma XC by blending with saline and physical manipulation (ie, massaging) of the products both increased the spread of all products within the anatomical planes (Figure 6D). However, despite equivalent manipulations, Restylane products Restylane-L and Restylane-Lyft appeared to remain distinctly within the planes they were injected in without spreading to adjacent tissues (Figure 6E).

## DISCUSSION

### Prospective Patient Injection Pilot Study

The 3D photogrammetry technology allows for high-level feature enhancement and anatomical evaluation and can be used to make precise volumetric measurements at injection sites to evaluate HA injection efficacy.^16-19^ Our research group has extensive experience with the implementation of precision metrics for facial aesthetic analysis, including the modality employed in this study. 3D photogrammetry technology was herein applied successfully to measure a small volume (<1 cc) of a soft HA filler within a precisely defined complex anatomical region over time. This prospective pilot patient injection study first quantifies precise and consistent volumetric measurements of the infraorbital tissue response to Volbella XC injections of the IOH, then analyzes volumetric treatment response within the context of PROs as well as validated infraorbital pretreatment severity scales.

Two studies have previously described 3D volumetric analyses of the IOR. In an attempt to assess the effects of gravity on the midface in individuals with prior facial dermal fillers, Ramesh et al reported filling of the IOR from sitting to supine, correlating to an increase in volume of the IOR by 0.59 mL, the tear trough by 0.22 mL, and the malar region by 1.2 mL.^20^ This study highlighted the importance of positioning to the morphology of the face as it relates to gravity, an observation that many clinicians have described anecdotally. Our work expanded on this premise by specifically addressing the contribution of orbicularis oculi muscle (OOM) activity on IOH and its treatment. The contribution of the tone of the OOM is well known to the surgical literature of the periorbit; however, its role in the treatment of the IOH through HA fillers has not yet been addressed. The novel data presented here with open eyes in forward gaze, closed eyes, and open eyes in upward gaze, reflect differential activity of the OOM relative to the IOH and its response to HA treatment. With the activation of the OOM, eyes closed, the IOH is diminished, in part because of the tightening activity of the OOM. In upward gaze and relaxation of the OOM in forward gaze, the IOH is more pronounced. These patterns are consistent after injection of the IOH with Volbella XC, as reflected in the TDF and EV measurements, conforming appropriate retention of OOM activity with treatment. At 90 days, volume maintenance was found to be best observed with OOM activation, potentially suggesting incorporation of Volbella XC into adjacent tissues while maintaining dynamic activation of the OOM for best long-term result. Together, these data support the recommendation of patient assessment and consultation with consideration of multiple eyelid positions as well as undercorrection of IOH with HA in the frontal gaze position. Specific attention should further be given to patients seeking neuromodulation of the inferior aspect of the OOM. Our analysis of differential eyelid positioning in the context of HA treatment of the IOH provides important insights for treatment and patient counseling recommendations for the IOR.

3D volumetric analyses of the IOR were also performed in the Phase III clinical randomized controlled trial in which patients with moderate or severe IOH were treated with Volbella XC.^6^ Participants in the treatment cohort received initial treatment (median 0.7 mL each side), an optional touch-up after 1 month (0.5 mL each side), and an optional repeat treatment at 12 months (0.65 mL each side). This study reported a mean volume increase from baseline of 0.857 mL (left) and 0.872 mL (right) at 3 months, and 0.733 mL (left) and 0.777 (right) at 12 months.^6^ Of note, in a subsequent publication of the same cohort, Fabi et al demonstrated Volbella XC's effectiveness to reduce IOH and improve overall satisfaction using validated PROs and pretreatment severity scales.^21^ While Fabi et al reported higher volumes in the IOR, we present an analysis of a more precise area of interest, with less overall product and no touch ups, corresponding more accurately to the typical clinical scenario of isolated IOH treatment. We believe the higher volumes reported previously were a result of (1) a larger area of interest, potentially capturing swelling outside of the IOR and the malar fat pads, (2) volume combined from the initial treatment and 1 month touch-up, and (3) more volume needed to treat moderate-to-severe IOH. After trialing different areas of interests, we selected a standard reproducible method to precisely measure the volume of the IOR at the specific sites of injection. Additionally, although in the previous prospective study on treatment for IOH, the authors reported a discontinuation rate of 6.25% to 10.7%, our study had a 100% response rate, highlighting the contribution of this work to volumetric and PRO results that clearly and consistently delineate the behavior of Volbella XC in this treatment area.^22^ Finally, our in depth analysis considers dynamic precise volumetric analysis through differential eyelid positioning to reflect the contribution of OOM activity during HA treatment of the IOR, as well as the important properties of TDF and EV.

In our volumetric analysis, the first 2 postinjection volume measurements allowed us to assess, for the first time in the IOR, important properties of the tissue response to a specific HA filler: (1) immediate postinjection response (TDF) and (2) the degree of HA tissue incorporation over the subsequent 14 days (EV). The observed TDF of Volbella XC in the IOH averaging 0.27 in repose, corresponds appropriately with the relatively low G′ of Volbella XC (271 Pa), and its design to be a soft and spreadable gel. Further, the high EV of Volbella XC after IOH treatment, averaging 95% in repose, suggests little postinjection swelling or inflammatory tissue response during the 2 weeks period postinjection. Together, these observations are consistent with a soft, natural correction of the IOH with minimal tissue stress and recovery.

Our volumetric temporal analysis revealed infraorbital HA volume maintenance was 70% to 81% at 30 days and 50% to 70% at 90 days. Although there was a slight decrease in undereye volume, these data may reflect additional tissue settling or HA product tissue incorporation during this time, as Volbella XC is designed to be a soft gel with low cohesivity. It was noteworthy, however, to observe that volume decrease over time was more prominent laterally, as depicted by the temporal color gradient. This observation may be explained by a higher HA metabolism in the dynamic lateral IOR (ie, orbicularis activation leading to lateral canthal lines) as these subjects did not receive lateral canthal line neuromodulation. Neuromodulation of the lateral canthal lines may thus promote HA volume maintenance in the lateral IOR.

We observed no statistically significant difference in TDF, EV, or volume maintenance by laterality, except when eyes were closed, with the left IOR losing slightly more volume than the right, as would be predicted anatomically with the left side of the face typically being more dynamic than the right. This finding suggests that laterality may influence volume maintenance of HA filler with asymmetric activation of the orbicularis oculi. However, because of the small sample size (*n* = 7), additional investigation is necessary to expand on this observation. Future studies with larger sample sizes with precise volume measurements are warranted to confirm the specific effect of laterality.

### Patient-Reported Outcomes

PROs revealed significant improvement in how participants rated their eyes and overall facial appearance over the span of 90 days postinjection. Although the maximum FDA-approved volume was 2.2 cc per side, our median volume per side was 0.88 cc, to reflect common treatment practice and maximize outcomes with a single treatment while minimizing adverse events. Our work thus supports the precise use of small amounts of soft HA gel in a single session to the IOH for positive patient outcomes. This finding is similar to Fabi et al's findings, where minor changes to the IOH postinjection had a major impact on patient satisfaction and quality of life.^21^ Significantly, our study also demonstrated significant positive change in participants’ perception of cheekbones at Day 90 compared with preinjection. This observation suggests that treatment of the IOH is beneficial to the appearance of the malar contour, perhaps by smoothing the cheek-lid junction through product integration into the tissue because of its low cohesivity, further augmenting patient satisfaction. Intriguingly, we observed the moderate correlation between TTRS and “Aging” to be statistically significant for the left IOR but not the right IOR, correlating with the loss of more volume in the left IOR, and corroborating studies that purport the left side of the face to be the more dynamic and “volumetrically weaker” side.

Our results indicate that the appearance of the tear trough, specifically, and asymmetry in facial features may impact patient perception on age, although the FACE-Q is a measure of the overall face and cannot be separated unilaterally. Together, this PRO analysis demonstrates significant clinical benefits for patients through the precise application of small amounts of Volbella XC for the treatment of the IOH, educating injectors on patient treatment benefits as well as expectations.

### Cadaveric Analysis

The cadaveric study highlighted the differential behavior of distinct HA filler properties injected in the infraorbital and malar regions. Tear trough and medial malar region filler injections resulted in precise anatomical placement along the infraorbital rim/ORL and in the deep medial cheek fat compartment, respectively. The Juvéderm Vycross products appeared to spread *across* anatomical planes more than the Restylane NASHA products. Our observed differences in HA behavior are consistent with known variances between these 2 HA technologies with respect to their cross-linking and rheology (G′, cohesivity, and flexibility). Juvéderm Vycross is a smooth gel while Restylane NASHA is particulate.^23-25^ Juvéderm is a monophasic material with no particles and its functionality is affected by the degree and type of cross-linking. Restylane is a biphasic material and has various particle sizes.^26^

Restylane has a higher elastic modulus (G′) than Juvéderm, resulting in a firmer and stiffer material that can explain the more targeted deposition of product with less distribution into surrounding tissues than the softer, less cohesive Juvéderm, as seen in our cadaveric models. G′, or ability to resist deformation modulus, is dependent on several factors including the type and degree of cross-linking, size of HA chains, and HA concentration. HA chains must undergo stabilization through cross-linking to prevent rapid in vivo enzymatic and oxidative degradation. In Juvéderm and Restylane filler products, HA is chemically cross-linked with 1,4-butanediol diglycidyl ether to produce a lasting dermal filler. The Juvéderm and Restylane products tested here have different concentrations of HA (15-20 mg/mL), and this concentration/weight includes both cross-linked and uncross-linked (free) hyaluronic molecules. Volbella (15 mg/mL) and Voluma (20 mg/mL) are highly cross-linked, whereas Restylane-L (20 mg/mL) and Restylane-LYFT (20 mg/mL) have a low percentage of cross-linking (∼1.2%). Increased degrees of cross-linking generally result in firmer and longer lasting gels, but in conjunction with pendant modifications can result in a softer gel and increased swelling.^22^

Manipulation of HA filler, including blending with saline and postinjection massage, can modify the natural rheological properties of the HA products, and in our cadavers appeared to spread the fillers medially and laterally *within* the injected anatomical planes, when compared with unmanipulated filler. It is important to note that despite the cadavers being fresh specimens, they do not allow for typical clinical hydrophilic swelling of the filler and its volumizing effect over time, nor exactly replicate spread and integration of the products as in dynamic living patients.

In summary, the results of the cadaveric study provide important anatomic descriptions about the behavior of diverse filler technologies commonly used in the treatment of IOH and the effects of manipulation techniques such as dilutional blending and massage. Confirmation of precise anatomical placement of the fillers and improved knowledge of their behavior in situ through this cadaver study may assist clinical practitioners’ selection of rheological properties of fillers, method of constitution, and manipulation technique depending on patient-specific characteristics for optimal outcomes in the delicate IOR.

### Limitations

Our pilot study has several limitations. First, during the 3D Vectra analysis, all measurements were based on the areas of interest, which were free-handed using the imaging software (VAM). This method potentially introduced modest variability between each subject and measurement. However, this was mitigated by following a standardized protocol created by the authors that allowed for consistent technique in aligning subject faces. We advocate for further research to validate its predictive power in evaluating change, utilizing additional imaging modalities (ie, ultrasound or CT imaging) as a control. Second, we were unable to account for confounding variables (BMI, age, etc) because of the small sample size and thus larger studies taking into account patient variables are warranted. Third, because Restylane products were not FDA approved for the treatment of the IOH at the time of our injection study, it was not possible to investigate a separate Restylane patient population for comparison. Additional follow-up studies are encouraged to assess these variables, as well as the long-term volumetric effect sand PROs of different HA fillers to the IOR. We propose additional data collected at 6 and 12 months and beyond to understand the metabolic process of HA in the IOH.

Although cadavers permit precise observation of anatomic details, cadaveric tissues do not behave like those of a live patient, which is important when considering HA fillers behave as biologic agents in response to dynamic tissues. There is minimal hydrophilic swelling of filler as would be in a live patient, and there is no spread or dynamic tissue integration of products. There is also no long-term follow-up. We were further limited by the number of cadavers available, prohibiting us from performing quantification of our observations; however, this was mitigated with the 3D volumetric study. Given our inability to compare products in vivo because of FDA drug regulations, our conclusions from the cadaver HA analysis are limited to those described as proof of concept.

## CONCLUSIONS

Small volume Volbella XC injection effectively augments undereye volume, as demonstrated by TDF and EV, to diminish IOH measured over a 90-day period with significantly improved PROs in the eye region and minimal patient recovery. Orbicularis oculi muscle activity and laterality may play an important role in treatment considerations. Advanced knowledge of the specific behavior of an HA filler, such as Volbella XC, in this precise anatomic region should be employed by clinical practitioners’ for the selection of specific HA products with appropriate rheological properties, method of constitution, and manipulation technique for optimal patient outcomes. Additional precise investigations of different HA products with larger sample sizes, additional patient characteristics and imaging modalities, as well as longer follow-up are recommended to optimize treatment of the delicate IOR.

## Supplemental Material

This article contains supplemental material located online at https://doi.org/10.1093/asjof/ojae086.

## Supplementary Material

ojae086_Supplementary_Data
