# Supplementary figures and images for: Multimodal Pilot Evaluation of a Hyaluronic Acid Infraorbital Filler Using Precise, Multipositional, 3-Dimensional Imaging Quantification, Patient-Reported Outcomes, and Anatomic Cadaveric Assessments
Source: Aesthet Surg J Open Forum. 2024 Oct 7;6:ojae086. doi: 10.1093/asjof/ojae086 (PMC11635449; doi:10.1093/asjof/ojae086)

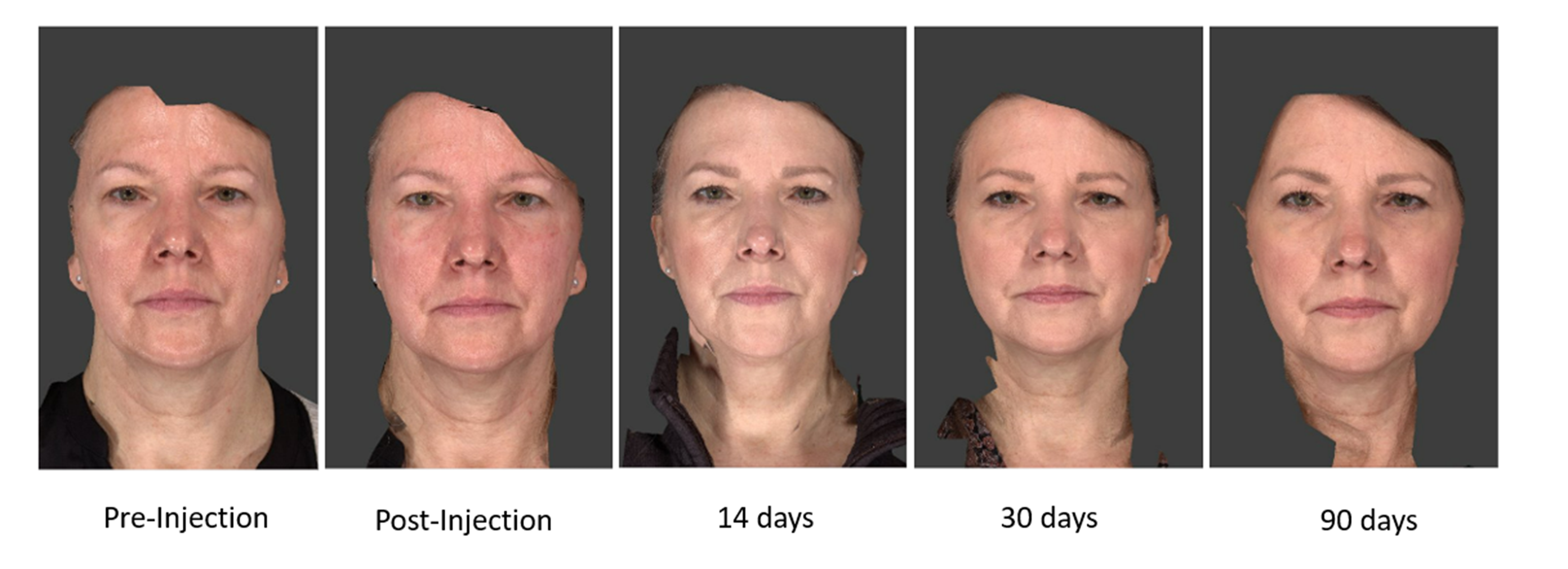

Supplement: ojae086_Supplementary_Data [file ojae086_Supplementary_Data.png]
